# Human Hair in the Stomach

**Published:** 1906-04

**Authors:** S. S. Rather

**Affiliations:** Jacksonville, Texas


For Texas Medical Journal.

### Human Hair in the Stomach.

BY S. S. RATHER, M. D., JACKSONVILLE, TEXAS.

Two years ago I was called to see Eunice Deaton, then 7 years old. Found stomach easily outlined by palpation and seemingly as hard as wood, vomiting everything that was put in stomach; very thin and anemic. This vomiting continued for ten days in spite of all kinds of treatment. Patient slowly improved and got up, but the hard stomach remained just the same. Every few months this same thing would happen with little variation from the first attack. As it was a puzzling case, I had consultation at various times with three other doctors; none were able to throw any light on the subject.

About January 1st she was taken with the severest attack she had ever had; in fact, nothing would pass out of stomach,—vomit medicine, diet, water, etc., as soon as swallowed. Told the mother to give half an ounce of castor oil every three hours for a day or two. After five doses tumor moved to lower bowels. Kept up oil one day more and the tumor moved to lower bowels, and found it to be her own hair—as much as any 10-year-old child has on its head. It was in three or four rolls two inches in diameter and eight inches long, matted and tangled in such a way that it was very hard. Had little or no feces mixed with it.

Patient got immediate relief and has improved rapidly ever since.

The mother now remembers that five years ago (the child was then 4) she was compelled to clip the child's hair short to keep her from eating it. The child acknowledged that she had a craving for it and would secrete herself and eat great quantities of it, but claims she has not eaten any since her hair was clipped.

I have given the true name of the patient. The Deaton family are a well-to-do family of the Craft neighborhood, and will vouch for all my statements.

